# Freestyle Libre‐Derived Metrics in Assessing Glycemic Control in Diabetic Dogs

**DOI:** 10.1111/jvim.70151

**Published:** 2025-06-18

**Authors:** Francesca Del Baldo, Antonio M. Tardo, Chen Gilor, Jocelyn Mott, Caterina Da Vela, Valeria Pergolese, Federico Fracassi

**Affiliations:** ^1^ Department of Veterinary Medical Sciences University of Bologna Ozzano dell'Emilia Italy; ^2^ Small Animal Internal Medicine, Department of Small Animal Clinical Sciences, College of Veterinary Medicine University of Florida Gainesville Florida USA

**Keywords:** ambulatory glucose profile, continuous glucose monitoring system, diabetes mellitus, glycemic variability, interstitial glucose

## Abstract

**Background:**

The FreeStyle Libre provides several metrics that are currently recommended for assessing glycemic status and guiding therapy in human medicine.

**Hypothesis/Objective:**

To evaluate the use of various FreeStyle Libre derived metrics for monitoring glycemic control (GC) in diabetic dogs.

**Animals:**

Eighty‐five client‐owned dogs with diabetes mellitus (DM).

**Methods:**

A retrospective review of medical records was performed to search for dogs with DM on insulin treatment and monitored with FreeStyle Libre. To clinically assess GC, the Agreeing Language in Veterinary Endocrinology diabetic clinical score was used (ALIVE‐DCS). Metrics evaluated were: percent time in range (TIR%), percent time above range (TAR%), percent time below range (TBR%), mean glucose (MG), percent coefficient of variation (CV%).

**Results:**

TIR%, TAR%, and MG were correlated with the ALIVE‐DCS (rs = −0.35, *p* = 0.02; rs = 0.31, *p* = 0.038; rs = 0.36; *p* = 0.016, respectively). The CV% was correlated with MG (rs = −0.70, *p* < 0.0001). CV% was higher in dogs experiencing low IG values compared to dogs that did not (44% [19–65] vs. 28% [8–67]; *p* < 0.0001). Dogs with optimal GC had significantly lower MG (240 [108–411] vs. 290 mg/dL [155–478]; *p* = 0.006) and TAR% (48% [0–93] vs. 64% [12–100]; *p* = 0.006) and significantly higher TIR% (49.5% [7–100] vs. 35.0% [0–85]; *p* = 0.009) compared with dogs with sub‐optimal GC.

**Conclusions and Clinical Importance:**

FreeStyle Libre derived metrics, particularly TIR%, TAR%, MG, and CV%, have potential utility in assessing GC in diabetic dogs.

**Abbreviations**
AGP: ambulatory glucose profile
ALIVE‐DCS: Agreeing Language in Veterinary Endocrinology diabetic clinical score
CGMS: continuous glucose monitoring systems
CV%: coefficient of variation
DM: diabetes mellitus
IG: interstitial glucose concentration
MG: mean glucose
TAR%: percentages of time above range
TBR%: percentages of time below range
TIR%: percentages of time in range

## 1 Introduction

In recent years, glucose monitoring has been revolutionized by the development of continuous glucose monitoring systems (CGMS), wearable minimally‐invasive devices which measure interstitial glucose concentration (IG) continuously for several consecutive days to weeks [1]. These devices reduce the need for repeated venipuncture and greatly increase the amount of information regarding glucose fluctuations and trends. These CGMS devices are generally well tolerated, easy to apply, and recent research has demonstrated their potential role in managing diabetes mellitus (DM) in dogs [1, 2, 3, 4, 5, 6, 7, 8, 9, 10]. The FreeStyle Libre (Abbott) is currently the most commonly studied CGMS in veterinary medicine. With this device, the IG data are transferred from the sensor to the reader (either a FreeStyle reader or a capable smartphone) when the reader is in close proximity to the sensor. Data are transferred automatically to a cloud‐based application (LibreView system) from the smartphone or can be uploaded from the FreeStyle reader to the same application. The LibreView System is a free, secure, cloud‐based diabetes management system provided by Abbott that enables remote data sharing with healthcare professionals. The system generates summary glucose reports from the uploaded IG data, including the Ambulatory Glucose Profile (AGP) and the Daily Log. The AGP report provides statistical metrics such as mean glucose (MG) and the percentages of time below range (TBR%), time in range (TIR%), and time above range (TAR%). Further, glycemic variability, expressed as percent coefficient of variation (CV%), is provided. Glycemic variability (GV) refers to intra‐day and inter‐day glycemic excursions including episodes of hypoglycemia and hyperglycemia. In people, GV is an indicator of glycemic control and is considered to be a risk factor for DM complications [11]. These metrics are derived from the glucose readings collected throughout the monitoring period, typically reported over a 14‐day cycle (but can be adjusted to include up to 90 days monitoring). In people, an international consensus panel recently published recommendations for use of AGP derived metrics in assessing glycemic status and guiding therapy [12]. These recommendations were endorsed by a number of international diabetes organizations and are nowadays emerging as additional glycemic targets and outcome measurements together with glycated hemoglobin [13]. Although FreeStyle Libre is increasingly used in diabetic dogs, its successful integration into routine clinical practice is still limited because of the lack of standardized interpretation of its data. To address this, it is essential to first evaluate the use of these metrics. Therefore, this retrospective study aimed to evaluate the use of various metrics readily available through the FreeStyle Libre device for monitoring glycemic control in diabetic dogs, comparing these metrics with the clinical assessment provided by the ALIVE Diabetic Clinical Score (ALIVE‐DCS). The hypothesis is that FreeStyle Libre derived metrics correlate with the ALIVE‐DCS in diabetic dogs, reflecting the relationship between glucose control and clinical assessment.

## 2 Materials and Methods

### 2.1 Case Selection Criteria

A retrospective review of medical records from 2 veterinary teaching hospitals (Veterinary Teaching Hospital of the University of Bologna, Bologna, Italy; Department of Small Animal Clinical Sciences of the University of Florida, Gainesville, Florida, USA) was performed to search for dogs with diabetes mellitus (DM) on insulin treatment and monitored with FreeStyle Libre between October 2020 and September 2024. Dogs were eligible for inclusion if at least 7 days of continuous glucose monitoring data were available, and if their medical records, including medical history, physical examination findings, and body weight, were complete. Both dogs with and without concurrent diseases were included. Dogs were excluded from the study when the medical records were unavailable or incomplete and when the IG data collection lasted less than 7 days because of the early detachment of the sensor.

### 2.2 Assessment of Glycemic Control

**FIGURE 1:**
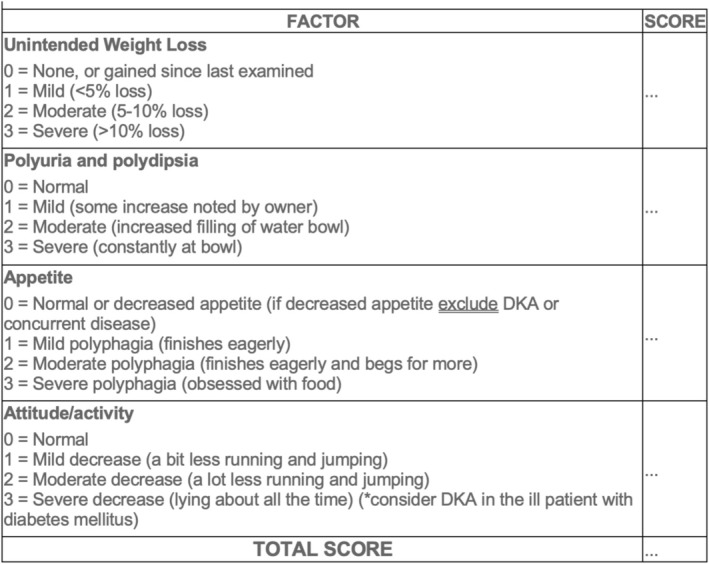
Agreeing language in veterinary endocrinology (ALIVE) Diabetic Clinical Score. Range total score: 0–12. The treatment aim is to have lowest score possible without unacceptably high risk of hypoglycaemia. Amended from: Niessen SJM, Bjornvad C, Church DB, et al. Agreeing Language in Veterinary Endocrinology (ALIVE): Diabetes mellitus—A modified Delphi‐method‐based system to create consensus disease definitions. Vet J. 2022;289:105910.

To clinically assess glycemic control, the Agreeing Language in Veterinary Endocrinology (ALIVE) diabetic clinical score was used (ALIVE‐DCS; Figure 1) [14]. This clinical score included consideration of the following variables: weight loss, presence of polyuria and polydipsia, appetite, attitude/activity. For each variable, a score was assigned as follows: 0 = normal/none, 1 = mild, 2 = moderate, and 3 = severe. Total clinical score (0 = ideal to 12 = poor) was obtained by summing the scores for each variable. According to the ALIVE‐DCS, dogs were categorized into two groups: optimal glycemic control (score 0) and sub‐optimal glycemic control (score > 0).

### 2.3 FreeStyle Libre Derived Metrics

The IG measurements were acquired using two generations of the FreeStyle Libre system (i.e., FreeStyle Libre 1 and FreeStyle Libre 2), validated for use in dogs (FreeStyle Libre, Abbott Laboratories Ltd., Chicago, Illinois) [5]. The detection limits of the sensors differ slightly, with FreeStyle Libre 1 measuring from 20 to 500 mg/dL and FreeStyle Libre 2 from 20 to 400 mg/dL. The following FreeStyle Libre derived metrics extrapolated from the AGP reports were evaluated: TIR% (percentage of time glucose within 70–250 mg/dL), TAR% (percentage of time glucose above 250 mg/dL), TBR% (percentage of time glucose below 70 mg/dL), MG and CV%. All these metrics are automatically calculated by the FreeStyle Libre application, considering the IG values detected during the entire functioning period of the sensor. For each dog, a single evaluation period was considered, and the extrapolated metrics referred to the entire functioning period of the sensor (a minimum of 7 days and a maximum of 14 days). The target ranges used were determined based on the current goals of diabetic treatment in dogs [15]. The glucose concentration ranges for TIR%, TAR%, and TBR% were set by accessing the “Report Settings” in the LibreView report viewer and adjusting the patient's threshold target ranges.

### 2.4 Statistical Analysis

Statistical analysis was carried out using commercial statistical software packages (GraphPad Prism 10, San Diego, California). Descriptive statistics were generated to characterize the study population. Continuous variables were presented as median and range (minimum and maximum value). The categorical variables were described with percentages. Correlations between ALIVE‐DCS and TIR%, TAR%, TBR%, MG, and CV% were evaluated using Spearman's rank correlation. Moreover, Spearman's rank correlation was used to evaluate correlation between CV% and MG. The correlation between ALIVE‐DCS and the different metrics was performed only in dogs in which the insulin dose has not been changed during the entire functioning period of the sensor (43 dogs). The FreeStyle Libre derived metrics relative to the entire monitoring period of the sensor were correlated with the ALIVE‐DCS measured at the end of the functioning period of the sensor. Mann–Whitney U test was used to compare CV% in dogs with and without concurrent diseases, as well as in dogs with and without low IG values detected by FreeStyle Libre, with low IG defined as at least one IG value < 70 mg/dL detected during the entire monitoring period. Moreover, Mann–Whitney U test was used to compare the FreeStyle Libre derived metrics in dogs with optimal glycemic control and dogs with sub‐optimal glycemic control. This study was designed as a preliminary investigation of these metrics, and as no prior information was available to allow for a meaningful sample size prediction, a formal power analysis was not performed. The level of significance was set at *p* < 0.05.

## 3 Results

### 3.1 Animals

A total of 85 dogs were enrolled. Forty‐four dogs were male, of which 24 were neutered, and 41 were female, of which 39 were spayed. The median age was 10 years (2–15 years) and the median body weight was 12 kg (3–45 kg). Mixed breeds (*n* = 24) were most common, followed by Poodle (*n* = 6), Labrador Retrievers (*n* = 5), English Setter (*n* = 5), Siberian Husky (*n* = 4), Yorkshire terrier (*n* = 4), Maltese (*n* = 4), West highland white terrier (*n* = 2), Border collie (*n* = 2), Lagotto Romagnolo (*n* = 2), Pomeranian (*n* = 2), Weimaraner (*n* = 2), Cavalier King Charles Spaniel (*n* = 2), Bolognese (*n* = 2), Pinscher (*n* = 2), Italian bloodhound (*n* = 1), Epagneul breton (*n* = 1), Doberman pinscher (*n* = 1), English pointer (*n* = 1), German pointer (*n* = 1), Golden retriever (*n* = 1), Dachshund (*n* = 1), English cocker spaniel (*n* = 1), Havanese (*n* = 1), Jack russell terrier (*n* = 1), Chihuahua (*n* = 1), Pug (*n* = 1), Terrier (*n* = 1), Bichon frisè (*n* = 1), Bichon havanais (*n* = 1), American Staffordshire terrier (*n* = 1), Bouvier des Flandres (*n* = 1).

Forty‐two dogs were treated with porcine Lente insulin, 17 with insulin glargine 300 U/mL, 9 with insulin detemir, 9 with insulin degludec 100 U/mL, 3 with a basal‐bolus protocol (glargine 300 U/mL + neutral protamine Hagedorn at the time of the meal), 2 with neutral protamine Hagedorn insulin, 2 with protamine zinc insulin, and 1 with glargine 100 U/mL. Sixty‐seven dogs received insulin twice daily and 13 dogs once daily. Of those that received insulin once a day (*n* = 13), 7 were on glargine 300 U/mL, 1 on protamine zinc insulin, and 5 on degludec. One or more concurrent diseases were documented in 46/80 (54%) dogs. The most common diseases included neoplastic conditions (12/46, 26%), naturally occurring hypercortisolism (9/46, 20%), chronic gastroenteropathy (5/46, 11%), exocrine pancreatic insufficiency (2/46, 4%) and hypothyroidism (2/46, 4%). The most common medications administered concurrently included trilostane (9/46, 20%), fenofibrate (8/46, 17%), ursodeoxycolic acid (7/46, 15%), systemic glucocorticoids (3/46, 7%), pancreatic enzymes (2/46, 4%) and levothyroxine (2/46, 4%). Twenty‐one glycemic control assessments were for dogs with newly diagnosed DM that had been receiving insulin treatment for ≤ 3 months, and 64 were for dogs with DM treated with insulin for > 3 months. The median insulin dose was 1.11 U/kg/day (0.08–4). Forty‐two dogs had their insulin dose changed during the selected monitoring period, while 43 did not. In dogs with and without concurrent diseases, the median insulin dose was 1.28 U/kg/day (0.08–4) and 1.08 (0.08–2.2) U/kg/day, respectively (*p* = 0.06). The median (range) ALIVE‐DCS was 1 (0–9). Glycemic control, as evaluated by use of the ALIVE‐DCS, was classified as optimal (= 0) in 26 dogs and sub‐optimal (> 0) in 59 dogs. The median TIR%, TAR%, and TBR% were 40% (0–100), 57% (0–100), and 1% (0–20), respectively. The median CV% was 38% (8–67). Forty‐five dogs had low IG values (IG < 70 mg/dL) recorded by FreeStyle Libre during the selected monitoring period. Eight dogs showed suspected episodes of clinical hypoglycemia. Of these, six had low (< 70 mg/dL) IG values detected by FreeStyle Libre, and two did not.

### 3.2 FreeStyle Libre Derived Metrics Data Analysis

The TIR%, TAR%, and MG were correlated with the ALIVE‐DCS (rs = −0.35, *p* = 0.02; rs = 0.31, *p* = 0.038; rs = 0.36; *p* = 0.016, respectively). In contrast, TBR% and CV% were not correlated with the ALIVE‐DCS (rs = −0.04, *p* = 0.80; rs = 0.10, *p* = 0.48, respectively). The CV% was correlated with MG (rs = −0.70, *p* < 0.0001).

The CV% was 42.6% (10.1–64.8) and 35% (7.6–67) in dogs with and without concurrent diseases, respectively (*p* = 0.14). Further, CV% was higher in dogs experiencing low IG values compared to dogs that did not (44% [19–65] vs. 28% [8–67]; *p* < 0.0001).

**FIGURE 2:**
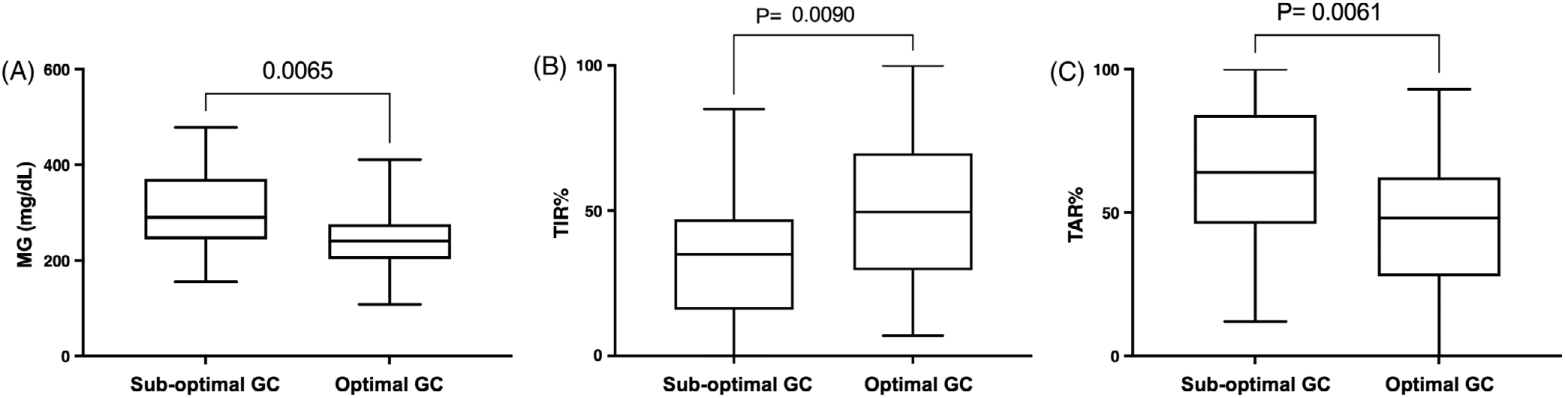
Box‐and‐whisker plots of MG (A), TIR% (B) and TAR% (C) in dogs with sub‐optimal (ALIVE‐DCS > 0) and optimal (ALIVE‐DCS < 0) glycemic control. The box represents the interquartile (25th to 75th percentile) range, the horizontal line in each box represents the median, and the whiskers represent the range. MG = mean glucose; TIR% = time in range; TAR% = time above range.

The results of the FreeStyle Libre derived metrics in dogs with optimal and sub‐optimal glycemic control are reported in Table 1 and Figure 2A–C. Dogs with optimal glycemic control had significantly lower MG (240 [108–411] vs. 290 mg/dL [155–478]; *p* = 0.006) and TAR% (48% [0–93] vs. 64% [12–100]; *p* = 0.006) and significantly higher TIR% (49.5% [7–100] vs. 35.0% [0–85]; *p* = 0.009) compared with dogs with sub‐optimal glycemic control. CV% and TBR% were not statistically different between the two groups.

**TABLE 1:** FreeStyle Libre (FSL) derived metrics in dogs with optimal (ALIVE‐Diabetic clinical score < 0) and sub‐optimal (ALIVE Diabetic Clinical Score > 0) glucose control.

FSL derived metrics	Dogs with optimal GC	Dogs with sub‐optimal GC	*p*
TIR%	49.5 (7–100)	35 (0–85)	0.009
TAR%	48 (0–93)	64 (12–100)	0.006
TBR%	2 (0–20)	1 (0–20)	0.3
MG (mg/dL)	240 (108–411)	290 (155–478)	0.006
CV%	39 (13–67)	35 (8–60)	0.40

*Note:* All values are presented as median (range). *p* < 0.05 was considered significant.

## 4 Discussion

The present study aimed to describe various metrics provided by the AGP report of the FreeStyle Libre system for monitoring glycemic control in diabetic dogs. Our findings suggest that TIR%, TAR%, and MG are significantly correlated with clinical assessments of glycemic control, as measured by the ALIVE‐DCS. Specifically, dogs with optimal glycemic control demonstrated higher TIR% and lower TAR% and MG compared to those with sub‐optimal control, reflecting the potential usefulness of these metrics in identifying effective insulin management. Despite the significant correlation between the ALIVE‐DCS and TIR%, TAR%, and MG, the strength of this correlation was only weak [16]. This is not unexpected, given the nature of the ALIVE‐DCS, which is largely based on the owner's subjective perception of clinical signs such as polyuria and polydipsia, appetite, and activity. The ALIVE‐DCS likely reflect the dog's clinical status in the days leading up to the assessment, rather than providing an estimate of the glycemic control over the previous 2 weeks. In contrast, the FreeStyle Libre‐derived metrics are calculated from continuous glucose data collected throughout the entire monitoring period. As such, they provide a more objective and comprehensive picture of glucose trends and fluctuations over time. The weak correlation might reflect the inherent difference between the subjective, short‐term perspective of the ALIVE‐DCS and the objective, long‐term glucose trends captured by the FreeStyle Libre. Additionally, owners may not always accurately report the severity or duration of their dog's clinical signs, leading to variability in the ALIVE‐DCS that does not directly correspond to the actual glycemic control. In contrast, FreeStyle Libre‐derived metrics are generated automatically by the system, without the potential for human error or bias. Also, many dogs in the study had concurrent diseases, which could have influenced the ALIVE‐DCS independently of glycemic control. Finally, the accuracy of the FreeStyle Libre system, although clinically acceptable, is not analytically perfect, which may have contributed to the observed weak correlation between the ALIVE‐DCS and FreeStyle Libre‐derived metrics.

In contrast, TBR% was not significantly correlated with the clinical score, suggesting that episodes of hypoglycemia detected by FreeStyle Libre may not always manifest in clinically observable clinical signs. This lack of correlation could be explained by the fact that the ALIVE‐DCS is not specifically designed to detect signs of hypoglycemia. Instead, the score primarily focuses on capturing signs caused by the occurrence of persistent hyperglycemia. Additionally, the accuracy of the FreeStyle Libre in the hypoglycemic range is not perfect, as studies in cats have shown a tendency to overestimate IG when BG is < 60 mg/dL [10]. It is possible that similar discrepancies may occur in dogs, potentially contributing to the lack of correlation between TBR% and the clinical score. These results underscore the potential limitations of the ALIVE clinical assessment alone in identifying hypoglycemic episodes and highlight the valuable role that metrics provided by the FreeStyle Libre system can play in offering additional insights. This data could be crucial for making more informed decisions regarding insulin dosing adjustments. In support of these findings, the clinical use of FreeStyle Libre has been demonstrated advantageous in people with DM in reducing hypoglycemic episodes [17]. In veterinary medicine, another study showed that the use of the FreeStyle Libre allowed more accurate identification of the glucose nadirs (79% vs. 41%) and hypoglycemic episodes (60% vs. 9%) as compared to the use of a traditional blood glucose curve as a monitoring method [6]. Given the limited number of suspected clinical hypoglycemia events in our study, larger‐scale studies are warranted to better understand the clinical significance of TBR% in the management of canine diabetes.

CV% is a marker of within‐day glucose variability (GV), which refers to the amplitude and frequency of variation from the average glucose level. In diabetic people, GV is an indicator of glycemic control [11]. A high GV is considered to be a risk factor for hypoglycemia, microvascular complications, neuropathy, nephropathy, retinopathy, stroke, and all‐cause mortality [11]. Although GV has not been extensively studied in dogs, the underlying physiological mechanisms affecting counterregulatory responses to hypoglycemia are believed to be similar between dogs and humans [18]. Based on this, it is reasonable to hypothesize that, as in humans, increased GV in dogs could contribute to a higher frequency of hypoglycemic events over time. Supporting this hypothesis, our results indicate that dogs with detected low IG values had significantly higher CV% compared to those without low IG values, suggesting a possible association between greater glucose variability and the risk of hypoglycemia in diabetic dogs. This indicates that CV% might be considered in identifying dogs at risk of hypoglycemia.

CV% was not correlated with the ALIVE‐DCS and, contrary to our expectation, it was negatively correlated with the MG. Several factors could explain this inverse correlation: (1) CV% detected by the FreeStyle Libre may be lower in dogs with marked hyperglycemia, as the sensor does not record glucose levels above 500 mg/dL (FreeStyle Libre 1) or 400 mg/dL (FreeStyle Libre 2), thus underestimating glucose variability in severely hyperglycemic dogs; (2) dogs with persistent hyperglycemia may exhibit fewer fluctuations in glucose concentrations, resulting in a lower CV%; (3) in dogs with lower median glucose levels, tighter glycemic control could result in more glucose fluctuations as adjustments are made to avoid hypoglycemia; consequently, dogs with better overall glucose control might show higher CV% due to greater fluctuations around a lower mean glucose level.

The lack of correlation between ALIVE‐DCS and CV% seems to be apparently in contrast to what is reported in human medicine, where high GV is associated with poor glycemic control and increased risk of diabetic complications [11]. A possible explanation for this finding might be that CV% not aligning well with the ALIVE‐DCS, as this score is based on subjective owner assessments at a single time point and does not reflect long‐term glucose trends. It is also worth noting that even dogs with an ALIVE‐DCS score = 0 had IG > 250 mg/dL for approximately 50% of the time. This highlights the limitation of owner‐based assessments, as they may fail to detect clinical signs and underestimate the severity of glycemic dysregulation. Therefore, further studies are needed to evaluate the correlation between CV% and clinical control in diabetic dogs.

Finally, while concurrent diseases are recognized contributors to GV [1], our study found no significant difference in CV% between dogs with and without concurrent diseases. However, the lack of statistical significance may be attributable to the limited sample size and to the limited range of the Libre. Notably, in dogs without concurrent diseases, the median CV% was less than 36%, which is the threshold typically used in humans to define the occurrence of glucose variability [13]. In contrast, dogs with concurrent diseases exhibited a median CV% greater than 36%. This discrepancy suggests that, although not statistically significant in our analysis, concurrent diseases may still play a role in influencing GV in diabetic dogs.

In this study, we evaluated FreeStyle Libre derived metrics in diabetic dogs with either optimal or sub‐optimal glycemic control. As these metrics have not been extensively characterized in diabetic dogs, the results offer initial insights into what constitutes “normal” or “abnormal” values for each metric, and can serve as a starting point for defining reference ranges in the management of dogs with DM. While our study provides valuable data, further investigations are needed to refine these metrics and establish standardized guidelines for their use in clinical settings, which will be critical for improving the management and treatment of dogs with DM. Incorporating these metrics into the monitoring of DM provides a more objective assessment of glycemic control, offering valuable insights into glucose fluctuations and trends both within and across days. This approach addresses the limitations of owner‐reported clinical evaluations, which are inherently subjective. As demonstrated in human medicine, the use of continuous glycemic metrics offers significant advantages, including a reduced risk of hypo‐ and hyperglycemia, improved long‐term glycemic markers such as HbA1c, and a lower incidence of diabetes‐related complications [12, 13]. Future studies should focus on standardizing these objective measures and integrating them with traditional clinical assessments to enhance diabetes management in veterinary practice.

A potential limitation of this study is the use of a rigid cut‐off to distinguish optimal from suboptimal glycemic control based on the ALIVE‐DCS score. While this approach was necessary to test associations with glycemic metrics, it might have led to the misclassification of some dogs with ALIVE‐DCS > 0 that were otherwise clinically well‐regulated. Additionally, the ALIVE‐DCS scoring system is designed as a clinical tool to assist decision‐making rather than as a validated classification method in research. It is also based on subjective assessments of glycemic control, which might introduce variability. It should be noted that the cut‐off applied in this study is not part of the ALIVE‐DCS score itself but was selected to allow for statistical comparisons. Beyond this limitation, several other factors should also be considered when interpreting our results. First, only a small number of dogs were included in the study. Second, the retrospective nature of the study may introduce selection bias, as only dogs with complete medical records and sensor data were included. Moreover, the variability in insulin treatment protocols across cases and insulin dose changes before inclusion could have influenced the results, and future studies should aim to standardize treatment approaches when evaluating CGMS‐derived metrics. As mentioned above, another limitation is the fact that dogs that are relatively well controlled clinically spend a lot of their time above the range reported by the Libre, which introduces bias into any comparison.

Finally, another limitation of this study is that several dogs were monitored with the FreeStyle Libre 2 system that has not been validated for use in diabetic dogs. However, since the FreeStyle Libre 2 uses the same sensing technology as the FreeStyle Libre 1 to measure IG, similar performance in terms of accuracy in diabetic dogs is expected.

In conclusion, our findings suggest that FreeStyle Libre derived metrics, particularly TIR%, TAR%, MG, and CV%, have potential utility in assessing glycemic control in diabetic dogs. These metrics provide insights into glucose trends that can complement traditional clinical assessments, thereby facilitating more precise insulin management.

## Disclosure

Authors declare no off‐label use of antimicrobials.

## Ethics Statement

As all dogs included in this study were presented for routine clinical rechecks, which are part of the standard care protocol for all diabetic dogs at our institutions, no additional stress or procedures were imposed on the animals to collect the data used in this study. For this reason, ethical approval was not required. Authors declare human ethics approval was not needed.

## Conflicts of Interest

The authors declare no conflicts of interest.
